# A Flowchart to Guide Emergency Physicians to Order Radiological Imaging in Pregnant Patients: Findings from an Emergency Department Questionnaire

**DOI:** 10.3390/healthcare13233138

**Published:** 2025-12-02

**Authors:** Fatih Cemal Tekin, Abdullah Enes Ataş, Fulya Köse, Demet Acar

**Affiliations:** 1 Department of Emergency Medicine, Konya City Hospital, 42020 Konya, Türkiye; dr_demetacar@hotmail.com; 2 Department of Radiology, Necmettin Erbakan University, 42080 Konya, Türkiye; aenesatas@gmail.com; 3 Department of Emergency Medicine, Bursa Yuksek Ihtisas Training and Research Hospital, 16310 Bursa, Türkiye; drfulyakose@hotmail.com

**Keywords:** emergency medicine, decision-making, diagnosis, medical education, lactation, obstetrics, pregnancy, radiology

## Abstract

**Highlights:**

**What are the main findings?**

- A significant majority (88.7%) of emergency department physicians report finding the management of pregnant trauma patients challenging and express a preference to avoid these cases.
- Physicians’ attitudes and decisions regarding imaging vary significantly based on their level of training and experience; Emergency Medicine Specialists and those with specific training are significantly more likely to order appropriate, immediate imaging for unstable patients regardless of gestational age.

**What are the implications of the main findings?**

- The widespread hesitation and practice variability among physicians, influenced by knowledge gaps and lack of guideline accessibility, may delay necessary diagnostics and compromise maternal–fetal outcomes.
- These findings demonstrate an urgent need for standardized education and practical, evidence-based decision-support tools; consequently, this study proposes a novel clinical algorithm (flowchart) to guide imaging decisions in emergency settings.

**Abstract:**

**Background/Objectives:** Emergency department (ED) physicians may hesitate to order medically indicated imaging (MI) for pregnant patients, potentially delaying or omitting necessary diagnostic procedures. This study aimed to assess the attitudes and practices of ED physicians regarding MI procedures in pregnant patients, considering their level of training. **Methods:** A total of 300 physicians participated, including ED general practitioners (*n* = 100), emergency medicine (EM) residents (*n* = 100), and EM specialists (*n* = 100). The first section of the questionnaire collected demographic data, professional experience, and previous training related to the subject. The second section included questions based on a modified version of the American College of Radiology (ACR) Appropriateness Criteria to assess attitudes and behaviors. **Results:** A majority (88.7%) of participants found the management of pregnant trauma patients challenging and expressed a preference to avoid such cases. A statistically significant difference was observed between physician groups regarding the approach to unstable pregnant patients, with EM specialists and residents more likely to disregard gestational age when deciding on imaging (*p* < 0.001). Physicians who had received relevant training were significantly more likely to advocate immediate imaging regardless of gestational age in unstable patients (*p* = 0.001). **Conclusions:** This study highlights the diversity and statistical heterogeneity in ED physicians’ attitudes and behaviors toward MI use in pregnant patients. These variations are influenced by training, clinical experience, and accessibility of updated guidelines. Enhancing education and standardizing procedural guidance may improve decision-making and ultimately reduce maternal and fetal morbidity and mortality. Based on these findings, we propose a clinical algorithm to support imaging decisions in emergency settings.

## 1. Introduction

The accessibility and use of medical imaging (MI) techniques have significantly increased recently. Consequently, the cumulative radiation dose to which individuals are exposed from these procedures has also risen, raising concerns about potential long-term effects [1,2,3,4]. While some physicians feel comfortable ordering and justifying MI procedures, most patients remain either unaware of or indifferent to the possible adverse outcomes [5,6,7]. However, during pregnancy, this dynamic reverses: patients show more awareness and concern, while doctors are more hesitant and anxious about using MI on MI in pregnant patients [8,9]. The fundamental concern specific to pregnancy is the potential teratogenic effects of ionizing radiation on the fetus. Although the impact on the pregnant individual receives comparatively less attention, there are still relevant health risks. Research on fetal and maternal radiation exposure is particularly challenging, and the limited availability of robust clinical data represents a major obstacle [10,11,12].

According to the traditional risk–benefit principle in medicine, the use of MI in pregnant patients should be justified through careful consideration of potential risks versus diagnostic benefits [13,14]. Although studies suggest that even full-body computed tomography (CT) scans do not exceed the established fetal threshold dose [15,16], clinical decisions involving imaging are not always straightforward. Trauma is still one of the main non-obstetric causes of maternal death, which is why MI procedures are so important for diagnosis. Thus, hesitation by physicians may delay timely and necessary diagnostic interventions, with potentially serious consequences for both mother and fetus [15,17,18,19]. In emergency departments (EDs), the need for rapid intervention in pregnant trauma patients further complicates the decision-making process for emergency physicians (EPs). Consequently, enhancing the knowledge, training, and confidence of EPs is essential for effectively managing these clinical scenarios.

Several studies in the literature have highlighted physicians’ concerns regarding the use of radiological imaging in pregnant patients. However, despite identifying this issue, no study has provided clear, practical, and implementable solutions to address these concerns. In this context, the present study takes an important step toward filling this gap from the perspective of Emergency Medicine. Additionally, by evaluating both the contribution of emergency medicine training to the management of trauma in pregnancy and its potential role in reducing the uncertainty surrounding diagnostic imaging, this research offers a unique contribution to the field and helps identify areas in need of further development. To address these concerns and elucidate physician decision-making processes, this study initially evaluates the attitudes and practices of emergency care providers, including Emergency Medicine Specialists (EMSs), Emergency Medicine Residents (EMRs), and Emergency Department Practitioners (EDPs), concerning the use of medical imaging in pregnant trauma patients. Thereafter, this study explores potential barriers that limit access to appropriate imaging for this vulnerable patient population and discusses strategies to improve evidence-based decision-making in emergency settings.

## 2. Materials and Methods

### 2.1. Study Design

**Figure 1:**
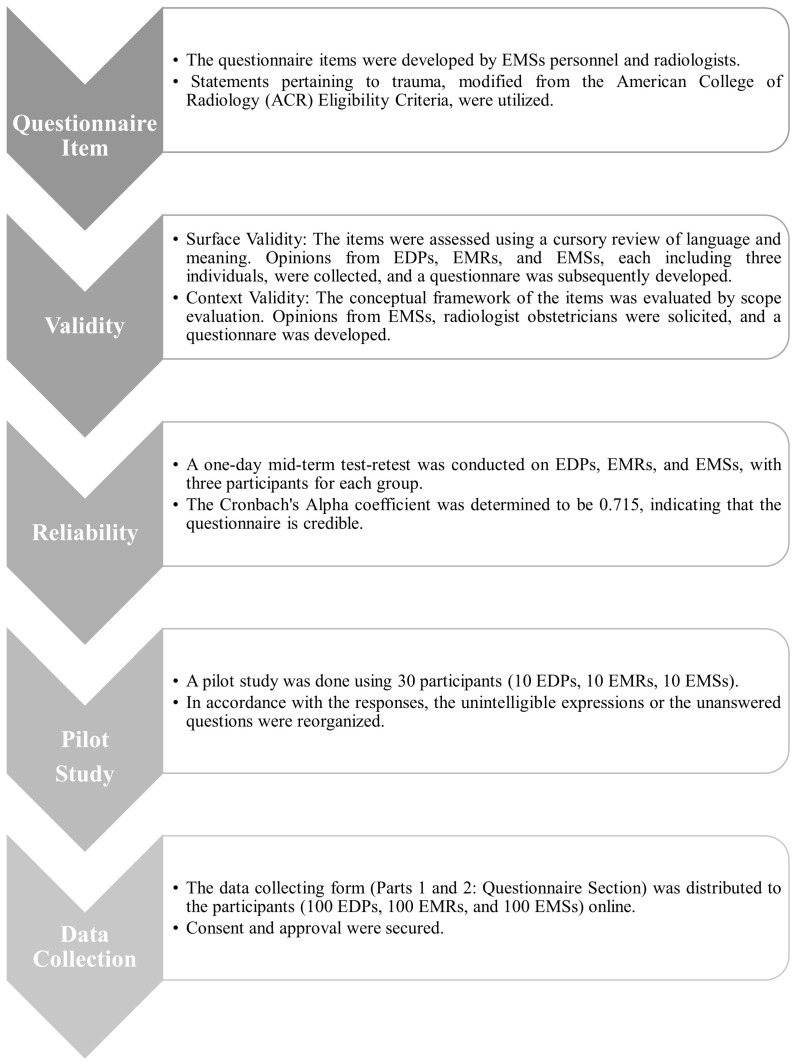
Protocol for developing a questionnaire. EMSs: Emergency Medicine Specialists; EDPs: Emergency Department Practitioners; EMRs: Emergency Medicine Residents.

This research was structured as a multicenter, cross-sectional investigation. Data were collected from October 2024 to January 2025 using a structured form consisting of two sections, which was administered to participants. The first section of the form included introductory information about the study and its objectives, followed by questions on sociodemographic characteristics, professional title, years of clinical experience, prior education or training on medical imaging (MI) in pregnant patients, and whether the participant reported discomfort in dealing with pregnant patients. The second section included items adapted from the American College of Radiology (ACR) Appropriateness Criteria [20], forming a questionnaire to evaluate participants’ attitudes and behaviors. Since physicians in the emergency department (ED) are more frequently exposed to trauma-related cases involving pregnant patients [18], the questions were framed with an emphasis on trauma scenarios. The complete form consisted of 26 items, including 14 structured or semi-structured questions in the first section and 12 attitude and behavior items (Q1–Q12) in the second. The questionnaire underwent validity and reliability testing (Figure 1), including a test–retest analysis that demonstrated over 95% consistency in responses.

### 2.2. Participants and Inclusion and Exclusion Criteria

The sample size was calculated based on unknown population parameters with a 90% confidence level, 5% margin of error, and an estimated proportion (*p*) = 0.5, resulting in a required sample of 272 participants. To ensure broader generalizability, ease of interpretation, and equal distribution across groups, the targeted sample was increased to 300 respondents equally distributed across three groups: 100 EDPs, 100 EMRs, and 100 EMSs. The inclusion criteria were agreement to participate and current employment as EDPs, EMRs, or EMSs in the emergency department. The exclusion criteria encompassed refusal to participate, incomplete questionnaires, or employment outside the ED.

Participants were selected using a simple random sampling method. First, the list of Ministry of Health–affiliated training and research hospitals was obtained. Physician names were identified through the official websites of these hospitals and subsequently assigned numerical codes. Based on this coding, participants were randomly selected using a simple random numbers table. Participants were recruited from EDs in training and research hospitals and were contacted up to three times to maximize response rates. Individuals who could not be reached or declined to participate were replaced with new participants selected using the same sampling procedure.

### 2.3. Questionnaire Scoring

In the questionnaire scoring system, responses that aligned with expected or guideline-based behavior were assigned 1 point, while all non-aligned responses received 0 points. The total questionnaire score was then compared based on participant characteristics such as professional title, clinical experience, and prior training.

### 2.4. Statistical Analysis

To assess whether participants’ training status influenced their consideration of gestational age when requesting radiological imaging, categorical variables were analyzed using the Chi-square (χ^2^) test, and comparisons involving more than two groups were conducted using multiway Chi-square analyses. Groups showing significant differences were further examined with Dunn–Bonferroni post hoc analysis.

The normality of continuous data distributions was assessed using Kolmogorov–Smirnov and Shapiro–Wilk tests, and homogeneity of variances was evaluated using Levene’s test. Statistical analyses examining the relationship between questionnaire scores and the variables of gender, marital status, and having children, as well as between questionnaire scores and participants’ responses regarding their own pregnancy experiences, the literature review habits, and course attendance, were conducted using Student’s *t*-test. Moreover, comparisons across multiple ordinal groups (the relationship between professional title, clinical experience, and questionnaire scores) were conducted using one-way analysis of variance (ANOVA), followed by either Tukey or Games–Howell post hoc tests depending on variance assumptions.

All statistical analyses were performed using IBM SPSS Statistics for Windows, Version 23.0 (IBM Corp.; Armonk, NY, USA), and a *p*-value < 0.05 was considered statistically significant.

## 3. Results

### 3.1. Participant Demographics

The median age of the participants was 32 years (range: 23–59). Among them, 198 (66%) were male and 102 (34%) were female. A total of 133 participants (54.4%) had less than five years of professional experience, while 167 (55.6%) had more than five years. The associations between participants’ gender, marital status, having children, professional title, and clinical experience and their questionnaire scores, as well as the statistical analyses examining the relationship between these parameters and questionnaire scores, are presented in Table 1.

The variations in questionnaire scores in relation to participants’ attitudes and training regarding pregnant trauma patients, as well as their own pregnancy-related experiences, along with the corresponding statistical analysis results, are presented in Table 2.

### 3.2. Attitudes and Training Regarding Pregnant Trauma Patients

A majority of emergency department (ED) physicians (266 participants: 88.7%) reported that managing pregnant trauma patients was challenging and indicated a preference to avoid encountering such patients (Table 2). Most respondents who reported this challenge were emergency medicine practitioners (EMPs: 97 participants, 36.5%). Additionally, 179 participants (59.3%) stated that they had not received formal training on managing pregnant trauma patients. However, 97.3% of all participants expressed interest in receiving such education and training. Among those who found managing pregnant trauma patients difficult, a significantly higher proportion desired training compared to those who did not (*p* < 0.001, χ^2^ = 12.228).

### 3.3. Questionnaire Scores and Participants’ Own Pregnancy Experiences

The relationships between participants’ responses to Section 1 of the data collection form and their total questionnaire scores are summarized in Table 2. A statistically significant difference was observed in total questionnaire scores across professional titles. Post hoc analysis revealed that the mean score of emergency medicine specialists (EMS: 7.35 ± 1.79) was significantly higher than those of other groups (EMS vs. EMR *p* < 0.001, I − J = −1.170; EMS vs. EMR *p* = 0.001, I − J = −0.880; EMR vs. EDP *p* = 0.471, I − J = −0.290). Additionally, statistically significant differences in questionnaire scores were found between individuals with less than one year of experience and those with 5–10 years of experience (*p* = 0.024, I − J = −0.885), as well as between those with less than one year and those with more than 10 years of experience (*p* = 0.012, I − J = −0.953).

### 3.4. Imaging Decisions in Unstable Pregnant Patients

In response to the question, “In which trimester would you order a radiation-based imaging test for an unstable pregnant trauma patient?”, 160 participants (53.3%) answered, “I would not consider gestational age.” The professional titles of physicians who selected this answer differed significantly from those who gave alternative responses (*p* < 0.001, χ^2^ = 29.116). Post hoc analysis indicated that this difference primarily involved comparisons between EMS and EMR, EMS and EDP, and EMR and EDP groups. Participants who had received training on this topic were significantly more likely to choose the option, “I would not consider gestational age in unstable patients” (*p* = 0.001, χ^2^ = 11.868). Furthermore, participants who selected this response had significantly higher total questionnaire scores (7.09 ± 1.78) compared to others (6.18 ± 1.76, *p* < 0.001, t = 4.446).

### 3.5. Imaging Modality Preferences

Regarding the question, “Which imaging modality would you choose first in unstable pregnant trauma patients with suspected head or neck trauma?”, 40% of participants preferred plain radiographs, 31.7% chose magnetic resonance imaging (MRI), and 24% selected CT or other imaging methods. Table 3 presents the responses to the questionnaire’s items categorized by educational attainment.

## 4. Discussion

In this study investigating medical imaging (MI) use in pregnant patients, the majority of emergency department (ED) physicians regarded this patient group as difficult to manage and expressed reluctance to encounter such cases. These attitudes likely stem from physicians’ concerns regarding the teratogenic effects of ionizing radiation during pregnancy, particularly in trauma scenarios. In clinical decision-making, especially in trauma cases, such hesitations may prevent pregnant patients from receiving the necessary diagnostic imaging. Numerous studies have advocated for the inclusion of radiologists in the decision-making process for myocardial infarction in pregnant patients; however, this methodology may not always be practical in emergency contexts due to time limitations and resource constraints [9,18,21].

The responses to the questionnaire revealed a substantial variation in physicians’ attitudes and behaviors toward imaging in pregnant patients, suggesting a significant knowledge gap. For certain questions (Q1, Q3, Q5, Q8, and Q9), the proportion of expected responses exceeded 70%; however, response rates were considerably lower for others, reflecting diverse practices and perspectives among physicians. Substantial variability emerged concerning the process of obtaining informed consent from unstable patients (Q2). While patient consent holds legal significance, it is fundamentally predicated on a relationship of trust. Ensuring patient autonomy requires comprehensive information exchange, whereby consent reflects mutual understanding rather than mere formality [22]. However, obtaining informed consent in ED settings can be challenging due to the urgency of treatment, time pressure, and patient-related limitations. In life-threatening situations, physicians may be compelled to proceed without formal consent, prioritizing maternal and fetal survival. Although ethically challenging, the establishment of clearer institutional protocols is essential to guide clinicians and reduce provider hesitation in such circumstances [23,24,25,26]. Moreover, these ethical and logistical challenges complicate imaging decisions and underscore the need for actionable solutions, including enhanced provider education, standardized protocols, and practical clinical algorithms to facilitate rapid, evidence-based decision-making in ED care.

Another issue approached with caution by ED physicians was contrast-enhanced imaging (Q4 and Q5). Studies indicate that low-osmolality iodinated contrast agents pose minimal fetal risk during CT; however, repeated exposure may necessitate neonatal thyroid screening [23,24]. Gadolinium-based contrast agents used in MRI, on the other hand, are not usually recommended. MRI, while generally considered safer due to the absence of ionizing radiation, may not be readily available or feasible in hemodynamically unstable patients requiring urgent imaging (Q10 and Q11) [25,26,27]. This highlights a need for clearer guidelines and improved availability of emergency protocols regarding contrast-enhanced CT and MRI use in unstable pregnant patients.

In questions related to fetal dose awareness (Q3 and Q9), participant responses aligned well with the expected knowledge and behaviors, reflecting satisfactory awareness among ED physicians. Although there is no universally defined safe threshold, radiation doses below 50 mGy generally do not cause deterministic fetal effects (Table 4) [16,28,29,30].

Technological advancements, especially during the COVID-19 era, have facilitated low-dose imaging protocols, such as ALADA (As Low As Reasonably Achievable), highlighting the importance of staff training in dose optimization and the establishment of dedicated imaging rooms for pregnant patients [31,32,33]. Table 5 presents types of examinations and the estimated fetal doses.

A striking finding of this study was the high proportion of respondents recommending temporary cessation of breastfeeding after contrast-enhanced imaging (Q6). Current guidelines suggest that even after gadolinium exposure, breastfeeding does not typically need interruption. However, some sources advise a 24 h cessation if the mother insists [17,34,35,36]. The variability in responses highlights a clear need for international consensus and multidisciplinary collaboration, especially when addressing postpartum imaging decisions. Efforts to raise awareness among ED physicians regarding this topic must be intensified.

Many ED physicians reported applying the same caution toward themselves or their partners as they would toward their patients regarding radiation exposure (Q12). While ethically commendable, this practice raises concerns that physicians may hesitate to prioritize appropriate diagnostics even in life-threatening scenarios. Such hesitation may jeopardize timely diagnosis and treatment in critical conditions like appendicitis or pulmonary embolism in pregnancy. The literature consistently emphasizes that the use of MI should be carefully considered but not avoided when clinically indicated. This finding reinforces the need for standardized, internationally endorsed emergency imaging protocols [6,37,38,39,40,41]. Our findings also indicated that EMS physicians performed better in balancing clinical decision-making in unstable patients, with higher total questionnaire scores (*p* < 0.001). This result suggests that EMS training significantly contributes to the effective management of pregnant patients in the ED settings. The important role of EMPs in the treatment process during high-risk presentations should not be overlooked. Participants who had received formal education on this topic demonstrated more appropriate attitudes and behaviors, further emphasizing the value of training.

Participants who found managing pregnant patients particularly challenging also exhibited significantly higher interest in education and training (*p* < 0.001), especially among EDPs. This supports the need for targeted educational interventions, such as webinars or in-person courses. Additionally, questionnaire scores were significantly higher among those who had completed relevant courses (*p* = 0.046) or read scientific literature (*p* < 0.001), indicating that education not only improves knowledge but may also reduce anxiety and enhance confidence in clinical decision-making. Lower scores among less experienced physicians (*p* = 0.01) further highlight that management of pregnant patients may be better handled by more experienced clinicians [41,42].

Pregnant patients frequently receive more detailed information about radiation risks than other adult or pediatric patients in the emergency department [43]. While it may be beneficial, it could also indicate the increased apprehension and anxiety among emergency department physicians regarding the risks of myocardial infarction during pregnancy, a trend documented in various studies [44,45]. Other studies similarly suggest that physicians tend to act more conservatively in referring pregnant patients for imaging, even when clinically necessary [41,46]. Our study confirms that such tendencies persist among ED physicians. The most concerning aspect, however, is the potential normalization of this reluctance. A significant contribution of this study is identifying this reluctance as an unresolved, critical challenge in emergency medicine. Furthermore, a notable gap exists in the literature addressing the specific roles and perspectives of ED physicians in managing imaging decisions in pregnant patients.

**Figure 2:**
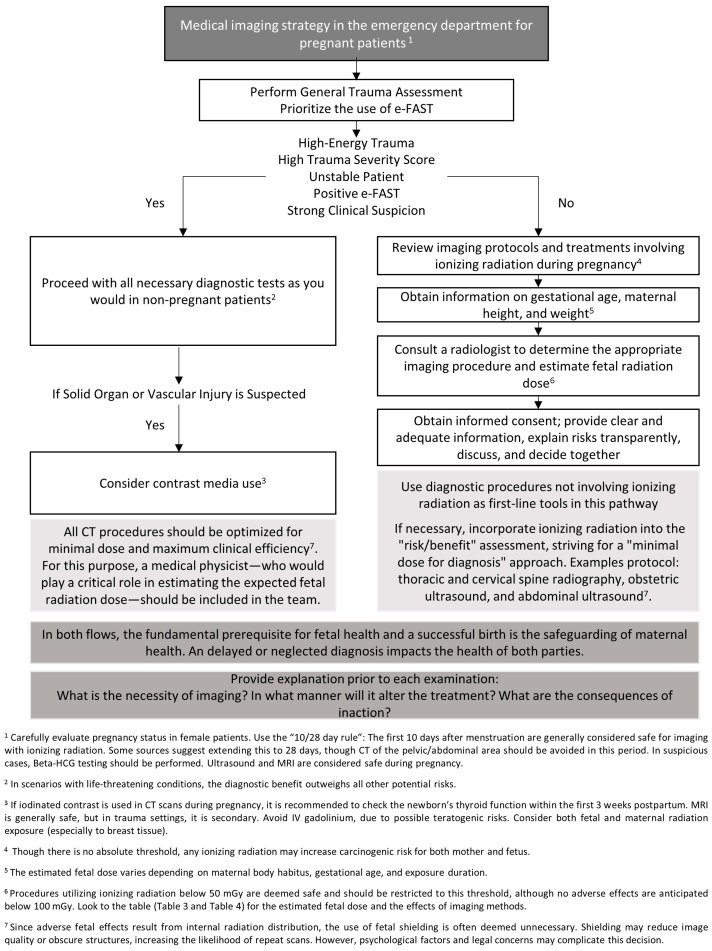
Imaging flow diagram for pregnant patients in the emergency department [17,25,31,45,47,48,49,50,51,52,53]. e-FAST: Extended Focused Assessment with Sonography in Trauma, CT: Computed tomography, Beta-HCG: Beta-human chorionic gonadotropin, MRI: Magnetic resonance imaging, IV: Intravenous.

This study reiterates a concern frequently reported in the literature: physicians often experience hesitation when requesting radiological imaging for pregnant patients, placing these patients at risk of being deprived of necessary diagnostic tools. Our findings underscore that this problem persists and highlight the urgent need for improvements in clinical practice. Bringing together variations in training, modality preferences, consent-related challenges, and post-procedural concerns, it becomes evident that emergency physicians would benefit from a structured decision-support tool. To address this need, we developed a clinical decision-making flowchart (Figure 2 (Refs. [17,25,31,45,47,48,49,50,51,52,53])) that synthesizes these complex considerations into a practical algorithm for emergency settings. Considering the identified knowledge gaps and the challenges faced by physicians, we believe that this algorithm will provide a meaningful contribution to the literature by supporting clinicians in making appropriate diagnostic imaging decisions for pregnant patients. We also hope that this effort will have a positive impact on reducing preventable maternal morbidity and mortality and offer tangible clinical benefits.

## 5. Limitations and Future Directions

A significant limitation of this study is that the number of participants can be considered small. However, there are similar studies conducted with fewer participants. An important limitation related to the sampling process is that the physician information available on some hospital websites was not up to date. Consequently, although the same sampling procedure was applied to identify subsequent participants, an unintended regional distribution may have occurred. Nevertheless, the study still achieved a participant pool that reflects substantial regional diversity. However, when interpreting the findings, it should be acknowledged that the results may be influenced by the fact that the study was conducted within a limited number of institutions and specific geographic regions. Although preliminary information may have been obtained for the proposed flowchart, the heterogeneity in attitudes and behaviors is important in terms of demonstrating the need for this algorithm. Awareness should be raised for studies to be conducted and guidelines to be developed in this direction.

## 6. Conclusions

By identifying these diverse perspectives and critical practice gaps, our study illustrates why achieving consensus on MI use in pregnancy is so urgently needed. Emergency department (ED) physicians exhibit substantial variability in their attitudes and behaviors regarding the use of medical imaging (MI) in pregnant patients. Similarly, the current literature lacks a unified approach and clear, standardized recommendations on this issue. One of the primary reasons for this inconsistency is the limited availability of scientific data. In addition to this knowledge gap, physicians’ hesitation toward MI use in pregnant patients stems from multiple concerns, including ethical, legal, and medicolegal risks, as well as anxiety over potential unknown consequences for the mother, fetus, and newborn. Diagnosing and managing traumatic emergencies in pregnant patients in the ED remains a critical and unresolved clinical challenge. Effectively addressing this issue may significantly contribute to reducing maternal mortality, particularly in trauma-related cases. Although clinical guidance from various specialties and organizations provides valuable insights, a clear and practical algorithm specifically tailored to emergency medicine remains a need. There is an urgent need for an international, multidisciplinary agreement to standardize imaging guidelines for pregnant patients, similar to the standardized resuscitation protocols that guide other critical care decisions. Developing accessible, evidence-based training programs will be equally vital to ensure physicians feel equipped to deliver optimal care under complex emergency conditions.

## Figures and Tables

**Table 1:** Comparison of demographic and general characteristics of participants with responses to questionnaire score.

Parameters		*n*	%	Questionnaire Score (Mean ± SD)	Statistical Result
Gender	Male	198	66	6.65 ± 1.90	*p* = 0.841 t = −0.201
	Female	102	34	6.70 ± 1.64	
Marital status	Married	188	62.7	6.60 ± 1.57	*p*= 0.615 t = −0.540
	Unmarried	112	37.3	6.71 ± 1.94	
Have child	Yes	128	42.7	6.79 ± 2.06	*p*= 0.314 t = 1.008
	No	172	57.3	6.58 ± 1.60	
Title	Emergency department practitioner	100	33.3	6.18 ± 1.64	*p* < 0.001 * F = 12.110 L = 0.616
	Emergency medicine resident	100	33.3	6.47 ± 1.80	
	Emergency medicine specialist	100	33.3	7.35 ± 1.79	
	<1	47	15.7	6.00 ± 1.58	*p* = 0.010 ** F = 3.556 L = 0.013
Experience (year)	≥1 and <5	86	28.7	6.52 ± 1.70	
	≥5 and <10	61	20.3	6.89 ± 1.56	
	≥10	106	35.3	6.95 ± 2.04	

SD: Standard deviation, t: Student’s *t*-test, L: Levene’s test, F: ANOVA. * Post Hoc Test: Tukey HSD; ** Post Hoc Test: Games Howell Min–Max: Minimum–Maximum. Statistically significant values are denoted in bold.

**Table 2:** The effect of participants’ own pregnancy experiences or those of their partners, literature review, and course attendance on survey scores.

Parameters		*n* (%)	Questionnaire Score (Mean ± SD)	Statistical Result
Emergency service management of traumatized pregnant patients is challenging and I would not like to encounter this patient group.	Yes	266 (88.7)	6.70 ± 1.79	*p* = 0.443 t = 0.769
	No	34 (11.3)	6.44 ± 2.28	
If you are a woman, did you experience a situation that necessitated radiologic imaging during your pregnancy? If you are a man, did you confront that same situation during your wife’s pregnancy? (*n* = 187)	Yes	29 (15.5)	6.55 ± 1.95	*p* = 0.897 F = 0.109
	No	158 (84.5)	6.71 ± 2.09	
(If you have encountered a situation that requires radiological imaging) Did you agree to have imaging? (*n* = 29)	Yes	25 (86.2)	6.76 ± 1.80	*p* = 0.155 t = 1.462
	No	4 (13.8)	5.75 ± 2.63	
Have you read any books or scientific literature on trauma management in pregnant women and the use of imaging methods?	Yes	117 (39.0)	7.16 ± 1.68	***p* < 0.001** t = 3.871
	No	183 (61.0)	6.35 ± 1.83	
Have you taken courses on trauma management in pregnant women and the use of imaging methods?	Yes	82 (27.3)	7.12 ± 1.80	***p* = 0.007** t = 2.693
	No	218 (72.7)	6.50 ± 1.79	
Have you attended a course or a certified program on trauma management in pregnant women and the use of imaging methods?	Yes	23 (7.7)	7.39 ± 1.58	***p* = 0.046** t = 2.003
	No	277 (92.3)	6.61 ± 1.82	

SD: Standard deviation, t: Student’s *t*-test, L: Levene’s test, F: ANOVA. Statistically significant values are denoted in bold.

**Table 3:** Attitude and behavior questionnaire items and distribution of responses.

Questions and Distribution			Agree	Disagree
Q1	In traumatizedtraumatised, unstable pregnant patients, laboratory tests should be expected first after physical examination.	EDPs (*n*)	68	32
		EMRs (*n*)	44	56
		EMSs (*n*)	72	28
		Total (*n*, %)	140, 46.3	160, 53.3
Q2	Informed consent must be obtained in traumatized, unstable pregnant patients, prior to imaging procedures involving ionizing radiation.	EDPs (*n*)	91	9
		EMRs (*n*)	88	12
		EMSs (*n*)	72	28
		Total (*n*, %)	251, 83.7	49, 16.3
Q3	In pregnant patients, it is important to order examinations based on the estimated fetal dose of the radiologic imaging procedure.	EDPs (*n*)	74	26
		EMRs (*n*)	80	20
		EMSs (*n*)	89	11
		Total (*n*, %)	240, 80.0	60, 20.0
Q4	In traumatized pregnant patients, if there is thoracic or abdominal trauma, CT imaging with IV iodinated contrast material should be performed.	EDPs (*n*)	21	79
		EMRs (*n*)	34	66
		EMSs (*n*)	39	61
		Total (*n*, %)	94, 31.3	206, 68.7
Q5	In traumatized pregnant patients, MR imaging with gadolinium can be performed.	EDPs (*n*)	27	73
		EMRs (*n*)	25	75
		EMSs (*n*)	33	67
		Total (*n*, %)	85, 28.3	215, 71.7
Q6	If the pregnant patient has given birth after diagnostic imaging with iodinated contrast media, I recommend that breastfeeding be discontinued.	EDPs (*n*)	75	25
		EMRs (*n*)	70	30
		EMSs (*n*)	59	41
		Total (*n*, %)	204, 68.0	96, 32.0
Q7	In traumatized pregnant patients, diagnostic imaging with a single dose of X-ray is not objectionable.	EDPs (*n*)	39	61
		EMRs (*n*)	44	56
		EMSs (*n*)	59	41
		Total (*n*, %)	142, 47.3	158, 52.7
Q8	In traumatized pregnant patients, a single dose CT (brain, cervical, thoracic, abdominal) can be easily ordered if necessary.	EDPs (*n*)	81	19
		EMRs (*n*)	79	21
		EMSs (*n*)	82	18
		Total (*n*, %)	242, 80.7	58, 19.3
Q9	Previous radiologic imaging for another reason may increase the cumulative fetal radiation dose in pregnant women, and it is important to question this situation.	EDPs (*n*)	95	5
		EMRs (*n*)	94	6
		EMSs (*n*)	89	11
		Total (*n*, %)	278, 92.7	22, 7.3
Q10	In traumatized pregnant patients, I can easily recommend MRI regardless of the gestational week.	EDPs (*n*)	68	32
		EMRs (*n*)	70	30
		EMSs (*n*)	72	28
		Total (*n*, %)	210, 70.0	90, 30.0
Q11	If the traumatized pregnant woman is unstable or if there is evidence of severe trauma in the patient, it is important to choose CT as the first choice when choosing between MR or CT.	EDPs (*n*)	45	55
		EMRs (*n*)	63	37
		EMSs (*n*)	74	26
		Total (*n*, %)	182, 60.7	118, 39.3
Q12	In the presence of high clinical suspicion of Pulmonary Embolism in a pregnant patient or in the presence of DVT, I would not hesitate to order Pulmonary CT angiography for the diagnosis of Pulmonary Embolism.	EDPs (*n*)	44	56
		EMRs (*n*)	28	72
		EMSs (*n*)	38	62
		Total (*n*, %)	110, 36.7	190, 63.3

CT: Computed tomography, IV: Intravenous, MR: Magnetic resonance, MRI: Magnetic resonance imaging, DVT: Deep vein thrombosis.

**Table 4:** Fertilization week an ionizing radiation.

Period	Week After Fertilization	Estimated Threshold Dose	Effect of Ionizing Radiation
Gestational	0–2	50–100 mGy	Death of embryo or no consequence
	2–8	200 mGy	Congenital anomalies
		200–250 mGy	Growth restriction
Fetal	8–15	60–310 mGy	Severe intellectual disability (high risk)
		200 mGy	Microcephaly
		25 IQ loss per 1000 mGy	Intellectual deficit
	15–25	250–280 mGy	Severe intellectual disability (low risk)

Gy (gray): The new international system (SI) unit of radiation dose, expressed as absorbed energy per unit mass of tissue. The SI unit “gray” has replaced the older “rad” designation. 1 Gy = 1 Joule/kilogram = 100 rad. IQ: Intelligence quotient. Modified from ACOG Committee Opinion No. 723: Guidelines for Diagnostic Imaging During Pregnancy and Lactation [17].

**Table 5:** Type of examination and estimated fetal dose.

Dose Classification	Radiography	mGy	Computed Tomography	mGy	Nuclear Medicine
Dose classification Very low <0.1 mGy	Any extremity	<0.001	Head or neck	0.001–0.1	
	Cervical spine (two view)	<0.001			
	Chest (two view)	0.0005–0.001			
Low to moderate 0.1–10 mGy	Abdominal	0.1–3.0	Chest or pulmonary angiography	0.01–0.66	Low-dose perfusion scintigraphy
	Lumbar spine	1.0–10			Technetium- 99m bone scintigraphy
					Pulmonary digital subtraction angiography
High 10–50 mGy			Abdominal	1.3–35	
			Pelvic	10–50	
					^18^F-PET/CT (Whole body)

Gy (gray): The new international system (SI) unit of radiation dose, expressed as absorbed energy per unit mass of tissue. The SI unit “gray” has replaced the older “rad” designation. 1 Gy = 1 Joule/kilogram = 100 rad. ^18^F-PET/CT: Fluorine-18 positron emission tomography/computed tomography. Modified from ACOG Committee Opinion No. 723: Guidelines for Diagnostic Imaging During Pregnancy and Lactation [17].

## Data Availability

Data is unavailable due to privacy and ethical restrictions. Ethical approval and informed consent for sharing the study data with third parties were not obtained. However, the data are stored in private dataset. To request access to the data, please contact the corresponding author (e-mail: fatihcemal.tekin@sbu.edu.tr).

## Abbreviations

The following abbreviations are used in this manuscript:

ACR	American College of Radiology
Beta-HCG	Beta-human chorionic gonadotropin
CT	Computed tomography
ED	Emergency department
EDPs	Emergency department practitioners
EMSs	Emergency medicine specialists
EMRs	Emergency medicine residents
e-FAST	Extended Focused Assessment with Sonography in Trauma
Gy	Gray
IV	Intravenous
IQ	Intelligence quotient
MI	Medical imaging
MRI	Magnetic resonance imaging
Q	Question
